# Do heart rates of elite marathon runners exhibit room for drift? Implications for durability

**DOI:** 10.3389/fspor.2025.1571498

**Published:** 2025-04-14

**Authors:** Fuminori Takayama, Atsushi Aoyagi

**Affiliations:** ^1^ The National Coalition of Independent Scholars, Brattleboro, VT, United States; ^2^ College of Education, Psychology and Human Studies, Aoyama Gakuin University, Tokyo, Japan

**Keywords:** cardiovascular drift, durability, elite athletes, fatigue resistance, running economy

## 1 Introduction

Recent advances in marathon research have suggested that factors beyond the three classic physiological variables—maximal oxygen uptake (VO2max), fractional utilization of VO2max, and running economy—such as pacing and drafting strategies, environmental conditions, neoteric footwear, and physiological resilience, significantly influence race performance (1). In particular, Jones explained that endurance exercise performance is not solely a function of a runner's physiological status at the start line, but is also related to the runner's fatigue resistance or resilience to changes in the aforementioned indices during the race itself (1). Fatigue resistance and resilience are also expressed in terms of durability. Durability is defined as an individual's ability to resist and delay the decline in certain physiological parameters, both in terms of magnitude and time of onset, during prolonged exercise (2).

Regardless of the formal definition of “durability” (2), some studies have been conducted from a durability-like perspective in marathon races (3–9). Notably, in real marathon races, previous research has utilized the runner's heart rate (HR) and running speed to evaluate this concept. HR is a valid indicator of a runner's internal load (10), and deviations therein relative to the external load (running speed) serve as a practical measure for assessing durability during a race (4). For instance, if cardiovascular (CV) drift, i.e., when HR increases despite a constant running speed, occurs early in a marathon, it can be interpreted as a sign of low durability (1, 4, 11). Additionally, heat stress can exacerbate CV drift by increasing CV strain, with factors such as ambient and core body temperatures, hydration status, and exercise duration also playing a role (12).

This opinion paper summarizes previous research on runners' durability during marathons and highlights the necessity for durability studies, particularly at the international level to world-class marathon runners.

## 2 Durability from recreational to national runners

As previously noted, durability during a marathon has been assessed based on the relationship between HR and running speed, which is commonly referred to as the cardiac cost (3) or internal-to-external workload ratio (4). Among these two measures, cardiac cost, defined as HR divided by the running speed, serves as a potential index for CV drift (3, 5). Shimazu et al. investigated the relationship between cardiac cost and marathon performance in 14 male university student runners (finish times ranging from 2:28 to 4:49) (5). A notable strength of this study was the use of an incremental treadmill test prior to the race to assess physiological performance, specifically the first ventilatory threshold running speed. They found significant correlations between changes in cardiac cost in later race segments (0–5 km vs. 25–30, 30–35, and 35–40 km) and relative performance, which was defined as the average marathon race speed relative to the first ventilatory threshold running speed (*r* = −0.672, −0.671, and −0.661, respectively). This result suggests that excessive CV drift has a negative impact on relative performance. Another interesting study, conducted by Billat et al., examined 280 recreational runners (finish times ranging from 2:30 to 3:40) and observed that nearly 80% experienced a decline in running speed during marathons (6). Notably, HR began to increase around the halfway point of the race, preceding a noticeable decrease in running speed, which became apparent at approximately 26 km. More recently, Smyth et al. analyzed the internal-to-external workload ratio (essentially the same metric as the cardiac cost) in 82,303 runners (mean finish time: 3:46) (4). Two noteworthy findings were obtained from this large-scale study: first, the onset of decoupling was observed at an average distance of approximately 25 km; second, the relative marathon performance was significantly associated with both the magnitude and the onset of decoupling. The decoupling onset occurred at 33.4 and 19.1 km in the low (high performance) and high (low performance) decoupling groups, respectively.

Furthermore, maintaining an essentially even running pace is crucial for achieving superior marathon performance. Ideally, HR should peak near the finish line as the race concludes. A previous study involving 50 New York City Marathon participants (average finish time: 2:54) demonstrated that runners whose HR did not increase or decrease in the later stages of the race tended to exhibit a decline in running speed (7). In addition, although the finish time itself is not at the international level or world-class in standard athletes, some case studies have shown that an HR pattern characterized by peaking near the finish line is associated with superior performance in well-trained runners (8, 9). Specifically, these studies included a national-level female runner and a visually impaired marathoner, both of whom set personal bests during their respective races, including a world record at the time, which was considered world-class within the context of parasports.

Based on these findings, sustaining a stable HR at the target race pace, particularly up to the 25-km mark (approximately 60% of the marathon distance), appears to be a key strategy for achieving optimal performance in recreational as well as national-level runners.

## 3 Determining factors of durability

Before discussing the durability of elite marathons, we will attempt to explain the factors that determine durability. According to the abovementioned study by Shimazu et al. (5), runners with higher relative performance (higher durability) had a better running economy, as measured by oxygen cost, than did those with lower relative performance. Although the energy derived from oxygen can vary slightly depending on the relative contributions of carbohydrates and fats, oxygen cost is closely related to energy expenditure because runners generate energy based on oxygen consumption (13). Moreover, a recent study showed that runners with superior running economy predominantly retain slow muscle fibers, which have superior fatigue resistance (14). Considering the advantages of a superior running economy, it is reasonable to hypothesize that it partially contributes to high durability.

Another possible factor in durability is carbohydrate (CHO) availability. Clark et al. demonstrated that CHO supplementation (60 g/h) during a 2-h heavy intensity cycling exercise preserved critical power, as compared to supplementation with placebo (15). This preservation may be associated with higher muscle glycogen levels, elevated CHO oxidation rates, and stable blood glucose levels (15). Although running and cycling differ in their specific demands, CHO availability plays a key role in endurance performance in both sports (16). Therefore, during marathon running, CHO availability is considered a potential factor influencing runners' durability.

Other potential factors include resistance to muscle damage and autonomic nervous system responses (17), all of which may be interrelated. However, the extent to which each of these factors contributes to durability, and whether their relative importance differs according to sex or performance level, remain insufficiently understood.

## 4 Durability from international level to world-class runners

While numerous studies have evaluated durability during marathon races in recreational and national-level runners (3–9), data from international level to world-class runners—those running within 2:09 for men or 2:19 for women (18, 19)—remain scarce, leaving many scientific questions unanswered. Current evidence indicates that, before the widespread adoption of advanced footwear technology, a male athlete who held the half-marathon world record (0:58:23) for a prolonged period exhibited remarkably high maximal oxygen uptake (83 ml/kg/min) and superior running economy (oxygen cost: 150 ml/kg/km) (20). Despite these superior physiological attributes, his marathon performance (2:08:46) fell short of being classified as world-class (≤2:03:00). This discrepancy underscores the likelihood that additional non-classical variables, with durability as a representative example, contribute significantly to marathon performance.

Traditionally, the relative intensity of marathons is closely aligned with the first metabolic threshold (first ventilatory or lactate threshold), regardless of runner level (21). However, this assumption does not fully capture the nuanced relationship between exercise duration and intensity. Jones and Vanhatalo showed that the relative intensity of 12 male world-class marathon runners (finish times ranging from 2:03 to 2:08) was higher than the first metabolic threshold, which corresponded to 96% of the critical speed (equivalent to the second metabolic threshold) (22).

When considering durability or decoupling among world-class runners, the data pertaining to the HR of a pacemaker that supported the former world-record holder Eliud Kipchoge during his record-breaking marathon (2:01:09) presents intriguing information (23). The pacemaker, who maintained a world-record pace shortly after the 20-km mark, appeared to operate at near-peak HR from the early stages of the race. These data were likely derived from wrist-based photoplethysmography (PPG) and did not provide clear information about the pacemaker's potential maximum HR, while the report has not undergone a formal scientific peer-review process; thus, this information should be interpreted cautiously because of potential validity concerns. Nevertheless, this case highlights an exceptionally high relative intensity in these runners as compared to runners at recreational and national levels. Notably, decoupling becomes particularly pronounced in recreational to national runners during the finishing time of international and world-class runners, highlighting an intriguing disparity in durability.

**Figure 1:**
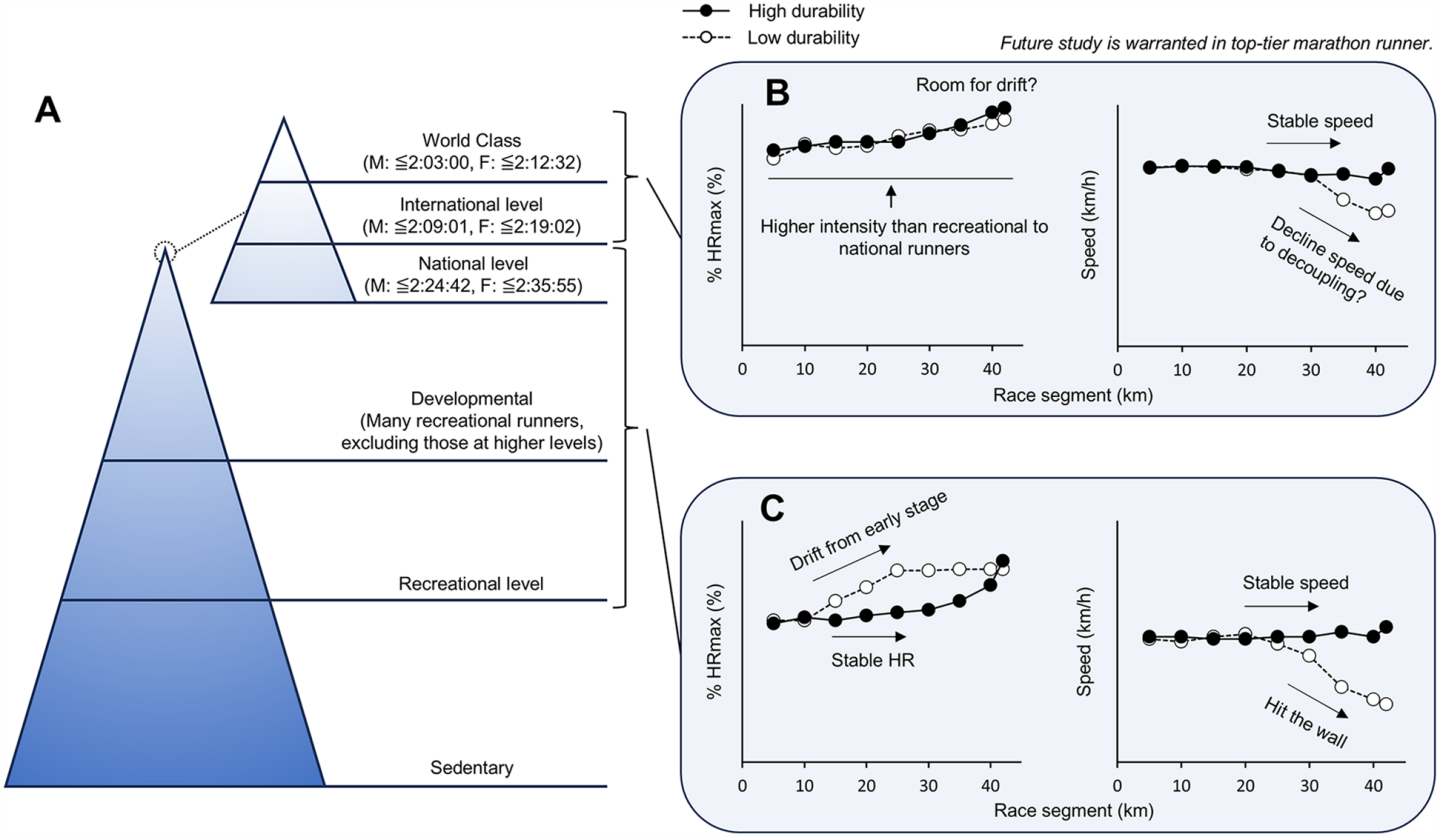
Summary of this opinion paper. **(A)** Hierarchical structure of marathon runners based on performance level, adapted from Burke et al. (18) and McKay et al. (19) using the world records at the time of writing as a reference. **(B)** Hypothetical heart rate and running speed during a marathon race in top-tier (elite) marathon runners. **(C)** Typically observed heart rate and running speed during a marathon race in recreational to national marathon runners.

Recreational and national-level runners typically run the early stages of a marathon race at <90% of their maximal HR (5), suggesting that they retain their reserve capacity, which allows for HR drift in the later stages of the race. In contrast, international and world-class runners run with little to no reserves from an early stage of the race. Consequently, if decoupling occurs in these runners, we suggest that it primarily results from a decrease in external load (running speed) rather than from internal limitations (Figure 1).

## 5 Discussion

Durability is a critical factor influencing marathon performance; however, it remains largely unexplored, particularly among international to world-class runners. The physiological demands of marathon running differ significantly between international to world-class runners and recreational to national-level runners (18). While competitive cycling has provided extensive insights into international and world-class athletes (24), comparable data are lacking for marathon running, leaving a significant gap in our understanding of the unique physiological demands faced by international to world-class runners. Recent advancements in wearable technology, such as PPG-based devices, offer practical tools for assessing HR during prolonged, high-intensity exercise and could enable new investigations in this area. As a preliminary step, future research may benefit from investigating male runners with finish times between 2:10 and 2:20 and female runners between 2:20 and 2:30. This approach is expected to enable a larger sample size and contribute to a more detailed investigation of unexplored aspects of durability during a race, particularly considering the lack of such data at this level before examining elite runners. Moreover, while the influence of sex differences is not addressed in this preliminary phase, findings from these male runner data may offer valuable insights into the durability of female international level to world-class runners.

Alternatively, a comprehensive research approach—incorporating field-based observations, controlled laboratory trials, case studies, and cross-sectional evaluations across different performance levels—could provide a more thorough understanding of the physiological factors influencing durability in marathon running.

However, without direct data from international and world-class marathon runners, the interplay among durability, decoupling, and marathon performance cannot be fully understood. Therefore, studies conducted under racing conditions involving international and world-class runners should be prioritized. Such research would not only address the existing knowledge gap, but can also provide actionable insights into optimizing performance strategies for top-tier marathoners.
